# High prevalence of haemosporidian parasites in Eurasian jays

**DOI:** 10.1007/s00436-024-08170-9

**Published:** 2024-04-16

**Authors:** Yvonne R. Schumm, Naemi Lederer-Ponzer, Juan F. Masello, Petra Quillfeldt

**Affiliations:** 1 Department of Animal Ecology & Systematics, Justus-Liebig University Giessen, Heinrich-Buff-Ring 26, 35392 Giessen, Germany; 2 Present Address: Department of Animal Behaviour, Bielefeld University, Bielefeld, Germany; 3 Present Address: Department of Biological Sciences, University of Venda, Private Bag X5050, Thohoyandou, 0950 Republic of South Africa

**Keywords:** Avian malaria-like pathogens, Blood parasites, Corvidae, Disease ecology, *Garrulus glandarius*

## Abstract

Avian haemosporidians are vector-borne parasites, infecting a great variety of birds. The order Passeriformes has the highest average infection probability; nevertheless, some common species of Passeriformes have been rather poorly studied. We investigated haemosporidians in one such species, the Eurasian jay *Garrulus glandarius* (Corvidae), from a forest population in Hesse, Central Germany. All individuals were infected with at least one haemosporidian genus (overall prevalence: 100%). The most common infection pattern was a mixed *Haemoproteus* and *Leucocytozoon* infection, whereas no *Plasmodium* infection was detected. Results on lineage diversity indicate a rather pronounced host-specificity of *Haemoproteus* and *Leucocytozoon* lineages infecting birds of the family Corvidae.

## Introduction

Vector-borne haemosporidian blood parasites (phylum Apicomplexa, order Haemosporidia), including avian malaria (genus *Plasmodium*) and malaria-like pathogens (genera *Leucocytozoon* and *Haemoproteus*), are widespread and infect a great variety of avian host species (Valkiūnas 2005). The application of molecular techniques for detecting and characterising haemosporidians is providing an increasingly growing and accurate picture of their host range, (host-specific) prevalence, distribution, and diversity (Rivero and Gandon 2018). The intensified research has revealed an extensive genetic diversity of haemosporidian lineages (> 4800 lineages, MalAvi 2023), which seems to be matched by an equally rich phenotypic diversity, such as lineages differing in their host range or within-host effects or virulence (Rivero and Gandon 2018; Ágh et al. 2022). At the avian host-species level, differences in host life history and behavioural traits, e.g. preferred foraging habitat or nest type, can influence rates of dipteran vector exposure, which can lead to heterogeneous infection probabilities across hosts (Fecchio et al. 2021). In a global study, including 141 avian families, those with the highest average *Leucocytozoon* infection probabilities belonged to the order Passeriformes, e.g. Paridae, Corvidae (Fecchio et al. 2021). Also, the highest *Plasmodium* lineage diversity across all continents was found in Passeriformes (Rivero and Gandon 2018). Nevertheless, in species of the order Passeriformes, many lineages and lineage-host relations likely were not found yet, particularly in hosts that have not been intensively sampled. For instance, there are 23 entries, covering around 60 individuals sampled in Africa, Asia and Europe, for Eurasian jays *Garrulus glandarius*, (Linnaeus, 1758) in the MalAvi database (274 entries in total for Corvidae), comprising seven *Haemoproteus*, two *Plasmodium* and five *Leucocytozoon* lineages (MalAvi 2023). The Palearctic-oriental distributed Eurasian jay is in Central Europe a resident, mainly forest-dwelling, open-nesting species. In some years, the species, predominantly individuals from Scandinavian breeding grounds, shows eruptive movements in autumn, which seem to be related to population density and the variation in acorn availability (Selås 2017). In this study, we aimed to (a) assess the Haemosporidia infection status in Eurasian jays from one forest population in Central Germany, (b) investigate the lineage diversity, and (c) compare our results with known lineages in Eurasian jays from other breeding sites.

## Material and methods

### Blood sample collection

We sampled 16 Eurasian jays from May to June 2020 and from May to July 2021. By limiting the sampling time to the spring–summer period, it was ensured that Eurasian jays from the local population and no migratory individuals were sampled. We captured the birds using walk-in traps placed in the Marburg Open Forest, a 250-ha managed forest, consisting of mixed stands dominated by common beech *Fagus sylvatica* and common oak *Quercus robur*, in Hesse, Central Germany (50°50′ N, 8°39′ E). In and around the forest site are smaller streams and temporary standing water bodies, which, amongst others, represent an ecological determinant of haemosporidian infections, as water bodies are necessary for the development of dipteran vectors (Ferraguti et al. 2018). We obtained the blood samples by puncture of the brachial vein (Table 1) and stored them on Whatman FTA cards (Whatman®, UK). Additionally, we prepared two blood smears per individual, which we fixed with methanol (100%) for 30 s and stained with Giemsa in a work solution prepared with buffer pH 7.0 (ratio 1:5) for 30 min.

**Table 1:**
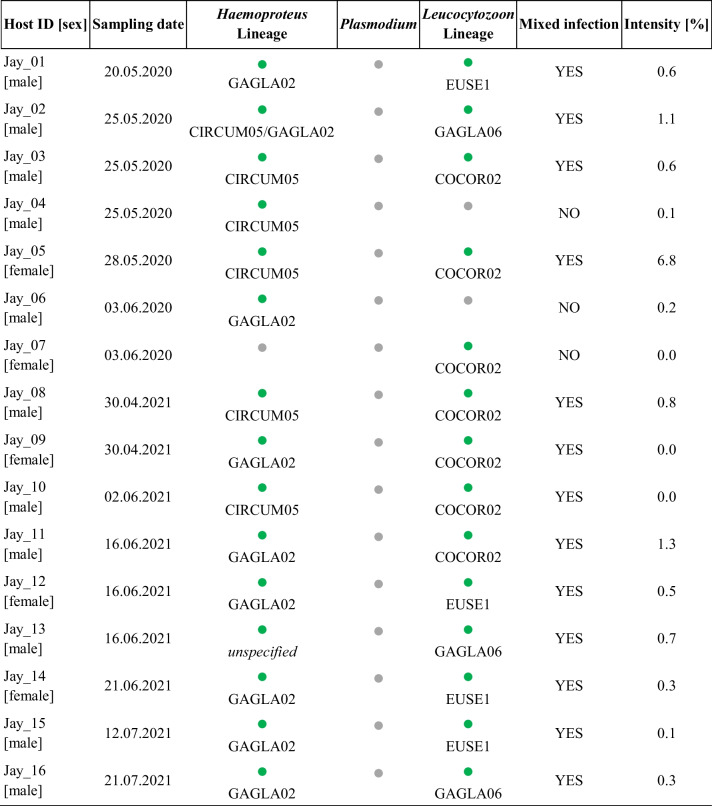
Avian haemosporidian infections in samples of Eurasian jays *Garrulus glandarius*

The infection status refers to nested PCR results (  = present infection,  = no detected infection), and the infection intensity (percentage of combined *Haemoproteus* and *Leucocytozoon* infected erythrocytes) to the blood smear counts

For DNA isolation, we cut a 3 × 3-mm piece of the samples in the FTA card to extract DNA (ammonium-acetate protocol; Martínez et al. 2009). We determined DNA concentration and quality using a NanoDrop2000c UV–Vis spectrophotometer (NanoDrop Technologies, USA).

### Parasite detection

We determined the presence or absence of avian haemosporidians through a nested PCR protocol targeting a 479-base pair (bp) region of the cytochrome *b* gene (cyt *b*; for detailed PCR protocol see Hellgren et al. 2004). In each PCR run, we included DNA from birds with known haemosporidian infection and deionised water as positive and negative controls, respectively. We rerun each sample resulting in a negative PCR reaction to confirm the parasite absence. We visualised the PCR amplicons using QIAxcel Advanced (QIAGEN) high-resolution capillary gel electrophoresis. Subsequently, we sent all PCR products of samples rendering a clear band during gel electrophoresis for Sanger bidirectional sequencing at Microsynth-Seqlab (Sequence Laboratories Goettingen GmbH, Germany). Using CLC Main Workbench 7.6.4 (CLC Bio, Qiagen, Denmark), we assembled and trimmed forward and reverse sequences. To identify lineages, we aligned the sequences with sequences deposited in the MalAvi database using BLASTN 2.3.0 + (Bensch et al. 2009). If the sequencing quality did not allow a clear determination, we repeated the PCR and sequencing. However, regardless of this, we could not clearly determine the lineage of one *Haemoproteus*-positive sample (Table 1).

**Fig. 1:**
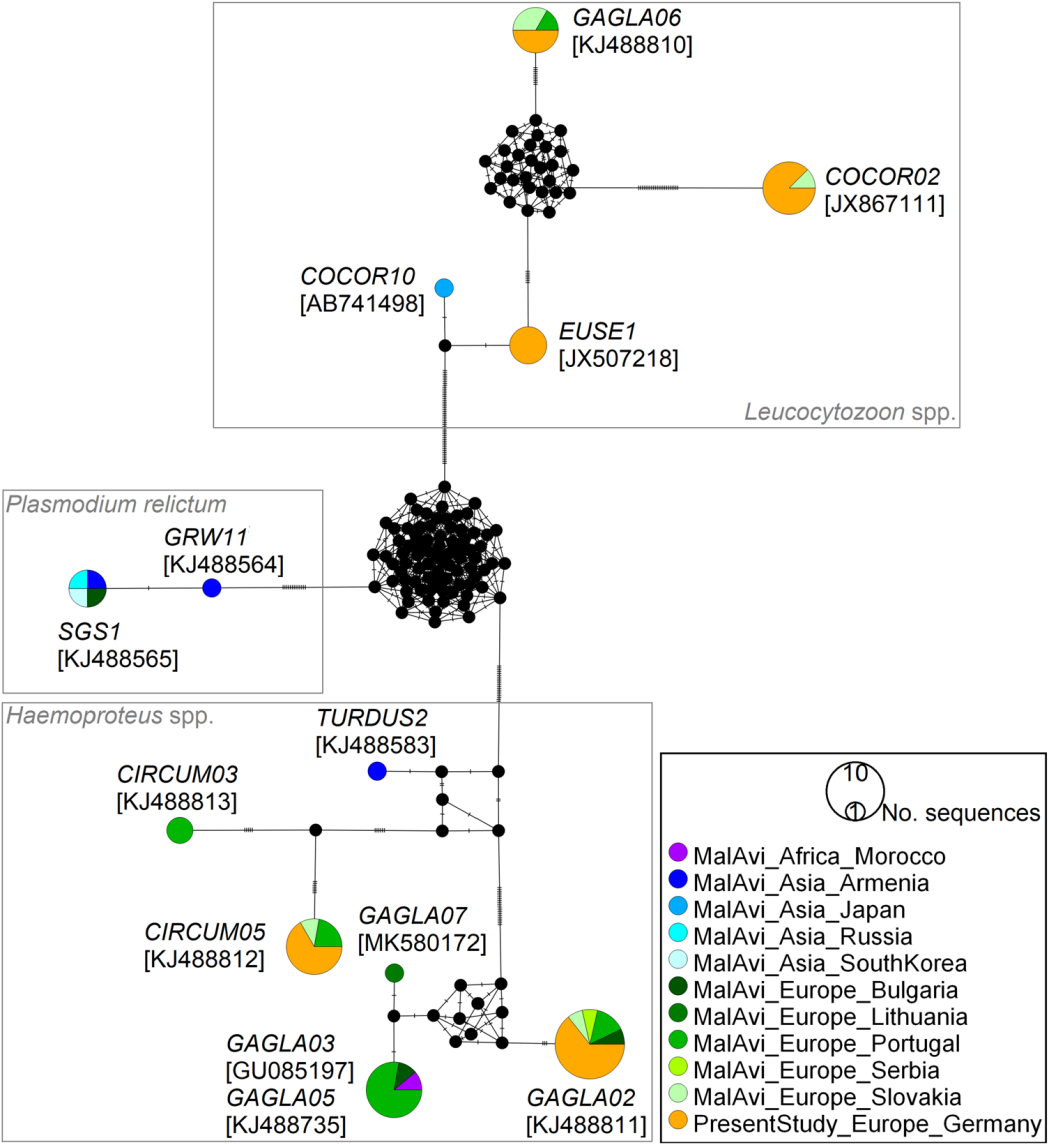
Median-joining network of mitochondrial cytochrome *b* lineages (476 bp, *n* = 60 sequences) of haemosporidian parasites found in Eurasian jays *Garrulus glandarius*. Circle size is proportional to the lineage frequency. Lineage names are noted at the associated circles together with one exemplary GenBank association number in parentheses. One hatch mark represents one mutation. Sample origins are represented by different colours, ‘MalAvi’ referring to sequences from other studies deposited in the MalAvi database (MalAvi 2023). Morphospecies names for GAGLA07 (*H*. *homopicae*) and TURDUS2 (*H*. *minutus*) are not provided within the figure

We constructed a haplotype network of haemosporidian lineages (Fig. 1), using the medium joining network method implemented in PopART 1.7 (Leigh and Bryant 2015). The network covers all sequences clearly assigned to a lineage from this study (*n* = 29) and sequences found in Eurasian jays deposited in MalAvi (*n* = 31). However, we had to exclude two sequences from MalAvi, corresponding to the lineages GAGLA01 and GAGLA04, due to being short (< 476 bp) and containing undetermined nucleotides.

We examined the stained blood smears at × 1000 magnification (light microscope PrimoStar Zeiss, Germany) for at least 10,000 monolayered erythrocytes to calculate the infection intensity based on the number of intraerythrocytic gametocytes of *Haemoproteus* and *Leucocytozoon*. No gametocytes of *Plasmodium* were detected during blood smear examination.

## Results and discussion

The overall infection rate in the Eurasian jay population was 100%, i.e. each individual showed a positive PCR result for at least one haemosporidian genus. Interestingly, we could detect no *Plasmodium* infections, while *Haemoproteus* and *Leucocytozoon* infections reached a high prevalence (94% and 88%, respectively), and often occurred as mixed infections (Table 1). Mixed infections, of either different species, genetic lineages or both in the same host, of haemosporidian parasites are common (Bernotienė et al. 2016). A recent study on Eurasian sparrowhawks *Accipiter nisus* showed that the prevalence of *Haemoproteus* was higher for hosts infected with *Leucocytozoon* and vice versa, i.e. a positive *Haemoproteus* × *Leucocytozoon* association (Svobodová et al. 2023). However, further investigations would be needed to elucidate if this represents a general pattern and an explanation of the high prevalence of mixed *Haemoproteus*-*Leucocytozoon* infections in our study.

*Plasmodium* infections (lineages GRW11 and SGS1, both *P*. *relictum*) have been found in a few Eurasian jays from Asia and Europe (Beadell et al. 2006; Dimitrov et al. 2010; Drovetski et al. 2014; Fig. 1), however other studies, including this one, did not find *Plasmodium* infections in this host species (Stanković et al. 2019; Šujanová et al. 2021). These differences might partly be due to a seasonal variation in the *Plasmodium* prevalence in temperate regions. For instance, Šujanová et al. (2021) report a higher-than-expected prevalence of *Plasmodium*-positive samples in autumn and Cosgrove et al. (2008) could show a clear pattern of seasonal variation of *P*. *circumflexum*, including an autumn peak. However, *P*. *relictum* showed a relatively stable seasonal pattern of prevalence (Cosgrove et al. 2008) and Neto et al. (2020) demonstrated the existence of a different seasonality of the same *Plasmodium* lineage between European countries (Bulgaria, Poland, Spain and Sweden). Generally, the entries on Eurasian jays in the MalAvi database included less than 10 jay individuals, except from the studies of Šujanová et al. (2021, *n* = 10, Slovakia) and Valkiūnas et al. (2019, *n* = 21, Lithuania), thus a general estimation of the variation in prevalence in this host is difficult with the available data. Both studies compared to our results observed a lower overall prevalence (30% and 5%, respectively).

Matching our result, a community analysis, including 29 avian species, from the same study site in Hesse found low *Plasmodium* infection prevalence (8%), compared to a higher *Haemoproteus* (68%) and *Leucocytozoon* (60%) prevalence (Strehmann et al. 2023).

The intensity of infection (parasitaemia) varied between 0 and 6.8%, however, most individuals (81%) had an intensity < 1.0% (Table 1). Generally, low-intensity infections (connected with mainly chronic infections) can persist in the hosts without direct visible, overt symptoms (Schoenle et al. 2017; Krams et al. 2022). This fits the observation that none of the sampled Eurasian jays showed any clinical signs of disease. However, low-intensity infections may have long-term detrimental effects on hosts, such as accelerated senescence or reduced reproductive success (Asghar et al. 2015; Schoenle et al. 2017).

We were able to assign the detected *Haemoproteus* infections to two lineages: GAGLA02 (prevalence: 56%, GenBank accession number: OR069477) and CIRCUM05 (38%, OR069478), whereby one Eurasian jay individual was infected with both lineages (Table 1). Both lineages already have been proved in Eurasian jays from other sampling locations in Europe (Fig. 1). Considering the lineage network, the GAGLA-*Haemoproteus* lineages cluster together (Fig. 1). The clustering lineages GAGLA02, GAGLA03 and GAGLA07 (*Haemoproteus* (*Parahaemoproteus*) *homopicae*) have so far only been detected in Eurasian jays, the lineage GAGLA05 also in another member of the Corvidae family, the common raven *Corvus corax* (Linnaeus, 1758; MalAvi 2023). This suggests a certain host-specificity of the found *Haemoproteus* lineages, which is in line with other findings proposing *Haemoproteus* to be rather host-specific (e.g. Ellis et al. 2020; Strehmann et al. 2023). The *Leucocytozoon* lineages GAGLA06 (OR069481) and COCOR02 (OR069479) were so far found in Eurasian jays and common ravens only, whereas the *Leucocytozoon* lineage EUSE1 (OR069480) up to now was detected in common ravens only (MalAvi 2023). We found four Eurasian jay individuals to be infected with EUSE1 (Table 1), constituting a new host-lineage interaction. The results show that similar to the *Haemoproteus* lineages, some *Leucocytozoon* lineages may be quite host-specific to Corvidae species. Therefore, this avian family, which has been rather poorly studied with respect to haemosporidian infections, might be a good model group for further research on haemosporidian host specificity.

## Data Availability

Sequences are deposited in GenBank (accession numbers OR069477/81) and will be submitted to the MalAvi database. The (raw) data of this article is freely available for download from PANGAEA at 10.1594/PANGAEA.961154, 10.1594/PANGAEA.961255, and 10.1594/PANGAEA.961493. A PrePrint can be found at bioRxiv: 10.1101/2023.06.20.545710.
